# Effect of non-surgical maxillary expansion on the nasal septum deviation: a systematic review

**DOI:** 10.1186/s40510-015-0084-y

**Published:** 2015-06-04

**Authors:** Tehnia Aziz, Kal Ansari, Manuel O. Lagravere, Michael P. Major, Carlos Flores-Mir

**Affiliations:** Division of Orthodontics, 5-528 Edmonton Clinic Health Academy, University of Alberta, 11405 87 Ave. NW, Edmonton, T6G 1C9 AB Canada; Division of Otolaryngology, 4071 Grey Nuns Hospital, Edmonton, T6L 5X8AB Canada

**Keywords:** Maxillary expansion, Nasal septum, Palatal expansion, Systematic review

## Abstract

Nasal breathing is a requirement for proper growth and development of the craniofacial complex. Inadequacy of the nasal airway from obstruction such as from nasal septal deviation (NSD) can affect craniofacial development. Further investigation of the possibility of rapid maxillary expansion (RME) correcting NSD would be valuable, considering the undesirable sequelae of NSD on nasal breathing, which can consequently affect craniofacial development. A systematic review of the effect of RME treatment on NSD was conducted. Electronic database searches were conducted until April 2015 using MEDLINE, EMBASE, Web of Science, Cochrane Database of Systematic Reviews (CDSR), Cochrane Central Register of Controlled Trials (CCTR), Cochrane Methodology Register (CMR), Database of Abstracts of Reviews of Effects (DARE), American College of Physicians Journal Club (ACP Journal Club), Health Technology Assessments (HTA), and NHS Economic Evaluation Database (NHSEED). MeSH terms used in database searches were ‘nasal septum,’ ‘palatal expansion,’ and ‘maxillary expansion,’ ‘orthodontic device,’ and ‘palatal expansion technique.’ The methodological quality of studies was reviewed using methodological index for non-randomized studies (MINORS). Only two studies were finally selected and reviewed. Both studies had significant methodological limitations. One study reported a significant straightening of the nasal septum in the middle and the inferior third of nasal cavity from RME in children aged 5 to 9 years. The other study reported no positional change in the nasal septum from RME in adolescent orthodontic patients. Thus far, the limited available (moderate risk of bias) evidence suggests a potentially positive effect on the nasal septum asymmetry during childhood, but no significant change in adolescence from RME in patients with NSD. The clinical significance of reported changes could be considered questionable.

**Electronic supplementary material:**

The online version of this article (doi:10.1186/s40510-015-0084-y) contains supplementary material, which is available to authorized users.

## Introduction

The nasal septum is an important functional and esthetic structure of the nose. It is responsible for regulating airflow through the nose while lending shape and support to the nasal dorsum and caudal aspect of the nose. Within the nasal cavity, a straight septum enables laminar airflow, allowing the inspired air to be warmed, cleaned, and humidified and thus optimized for gas exchange at the alveoli in the lungs. Conversely, a deviated nasal septum can contribute to various degrees of nasal obstruction and altered nasal respiration [1].

Nasal septal deviation (NSD) is defined as deviation of either the bony or the cartilaginous septum or both from the midline. Although, the earliest investigation reported 80 % of humans having some degree of septal deviation [2], more recent numbers in adults range around 65 % [3]. The prevalence range of NSD in neonates has been reported between 1 [4] to roughly 20 % [5]. In school-aged children (6–15 years), it was documented as 20 % when assessed on occipitomental projection radiographs, whereas the clinical diagnosis of NSD was made in approximately 10 % of the same cohort of children [6].

Nasal obstruction from a deviated nasal septum may cause turbulent nasal airflow precipitating in dryness and crusting of the nose, frequent nosebleeds, and recurrent sinusitis [7]. Furthermore, during developmental years, inadequacy of the nasal airway can necessitate chronic mouth breathing, causing moderate to severe maxillary constriction, and a vertical skeletal growth pattern characterized by long anterior lower face height, bilateral maxillary crossbite, high arched palate, low tongue posture, and incompetent lips [8, 9]. In addition, it has been hypothesized that nasal breathing is a requirement for proper growth and development of the craniofacial complex [10]. According to the functional matrix theory, nasal airflow is a continuous stimulus for lowering of the palate and for lateral maxillary growth, indicating a close relationship between nasal breathing and dentofacial morphology.

Rapid maxillary expansion (RME) is routinely used in orthodontic treatment to correct transverse maxillary constriction by opening of the midpalatal suture [11]. It works by separation of the two halves of the palatal bones across the median palatal suture due to a lateral force from the appliance [12]. Both the zygomatic and sphenoid bones of the cranial base are met with resistance during expansion. Therefore, the separation of maxillary bones occurs in a triangular manner, with the apex toward the nasal cavity and the base at the same level as the palatine process [13] resulting in more opening anteriorly than posteriorly [14]. Thus, one can extrapolate that there will be greatest improvement in the caliber of the anterior rate limiting nasal valve area from RME compared to other regions of the nasal cavity. Some studies have reported correction of septal deviation as an incidental finding from RME [15, 16]. To our knowledge, no review of the literature has been conducted to investigate this finding. Therefore, the purpose of this systematic review is to methodically analyze the available literature concerning the effects of RME on nasal septal deviation.

The specific PICOS question to be addressed is “In children or adolescent patients with a deviated nasal septum and a transversally constricted palate, does a nonsurgical palatal expansion produce a simultaneous improvement on the nasal septum position?”

## Review

### Methods

A review protocol was discussed, but it was not registered online.

Several databases were searched electronically with the help of a senior librarian specializing in health sciences database searches. The searched electronic databases were MEDLINE (from 1966 to the second week of April 2015, OVID), EMBASE (from 1974 to the second week of April 2015, OVID), Web of Science (from 1945 to the second week of April 2015, Thomson Reuters), Cochrane Database of Systematic Review (CDSR), Cochrane Central Register of Controlled Trials (CCTR), Cochrane Methodology Register (CMR), Database of Abstracts of Reviews of Effects (DARE), American College of Physicians Journal Club (ACP Journal Club), Health Technology Assessments (HTA), and NHS Economic Evaluation Database (NHSEED) until the first quarter of 2015.

The MeSH search terms used in database searches were ‘nasal septum’, ‘palatal expansion’, and ‘maxillary expansion’, ‘orthodontic device’, and ‘palatal expansion technique’ (see Additional file 1 for specific search terms and their combinations). These combinations of terms were identified with the help of a specialized health science librarian. No language limitation or year of publication limit was set.

Two authors (T.A. and K.A.) independently reviewed the title and abstracts of the database searches. Abstracts from human studies that discussed an orthopedic effect on the nasal septum from nonsurgical palatal expansion were included at the initial selection phase. The full text of all studies that appeared to meet the inclusion criteria were retrieved along with ones that had insufficient information in the abstracts to make a final decision regarding their inclusion. The references of retrieved articles were also manually searched for additional studies that could be included in the systematic review. The authors (T.A. and K.A.) independently assessed full articles obtained for inclusion in the systematic review, and any disagreement was settled through discussion until a consensus was reached.

In summary, the inclusion criterion was any type of clinical trial that evaluated objectively the orthopedic effect on nasal septum from nonsurgical palatal expansion procedures.

The following exclusion criteria were finally applied to the studies after retrieval of full text of articles:

1. No case reports
2. Studies that reported the presence of any concurrent sino-nasal pathology in their patient sample that would preclude visualization of the nasal septum before or after RPE treatment were excluded (examples of such conditions included, but not limited to, were septal perforation, enlarged turbinates and nasal polyps, etc.)
3. Studies that merely reported a visual change in NSD as an incidental finding and did not implement protocols to methodically measure nasal septum pre- and post-expansion were also excluded
4. Surgically assisted palatal expansion

Methodological scoring to assess the quality of included studies was also performed independently by two authors (T.A. and K.A.) through methodological index for non-randomized studies (MINORS) checklist [17]. Any disagreement in individual scores was settled through discussion until the final consensus was reached. Although an overall quality score was tabulated, it was established that the quality assessment would be considered in the discussion and data synthesis mostly through analysis of individual components.

### Results

**Fig. 1:**
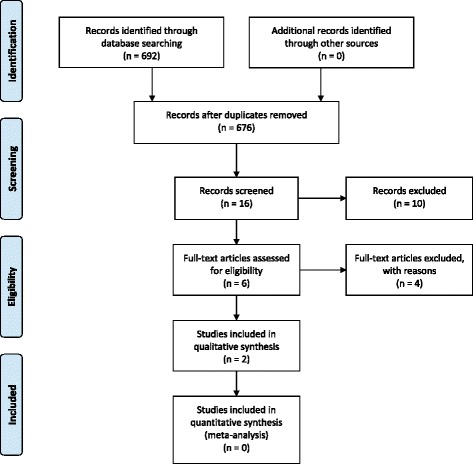
Flowchart of systematic review selection process

The flow chart of the electronic database searches and the final selection of studies to be included in the systematic review are outlined in Fig. 1 (numbers from the end of May 2014). Online searches resulted in six potential abstracts [15, 16, 18–21] after removal of duplicates that resulted from the overlap of studies between the electronic databases. Four studies [16, 18–20] were later excluded after a full review of the articles, and the reasons for their exclusion are listed in Table 1. This resulted in only two studies [15, 21] to be included in this systematic review. Key details of the included studies are listed in Table 2.

**Table 1:** Excluded studies and reason for their exclusion

Studies excluded	Reason for exclusion
Baydas et al. [18]	No mention of nasal septum evaluation
Schwarz et al. [19]	Only surgical RME discussed with respect to changes in nasal septum
Gray LP [20]	Reported a visual change in nasal septum from RME without employing methods to measure the change
Gray LP [16]	Reported a visual change in nasal septum from RME without employing methods to measure the change

**Table 2:** Characteristics of included studies

Study	Baseline characteristics of treatment group	Baseline characteristics of control group	RME protocol	Measurement of the nasal septum	Results
Farronato et al. [15]	*N* = 100 Ages 5–9 years (mean = 7.62 years, SD = 0.7) Nasal septal deviation (NSD) of more than 1 mm as seen on PA radiographs (amplitude of deviation)	*N* = 40 Ages 5–9 years (mean = 7.62, SD = 0.7) Not treated with RME Not clear if they presented with NSD	Hyrax expander 1 turn (0.25 mm) twice a day for 15 days	Amplitude of NSD measured on frontal/PA cephalograms as millimeter distance between midline axis of symmetry and deviated nasal septum. Measurements taken before appliance insertion (T0), at appliance removal (T1) and 6 months after appliance removal (T2)	94 % reduction in amplitude of NSD from RME in the middle and lower third of the nasal cavity from T0 to T2
Altug-Atac et al. [21]	*N* = 10 Ages 13–17 years (mean = 15 years) Nasal septal angle (from midsagittal plane = 1.05° (SD = 0.91))	*N* = 10 Ages 13–17 years (mean = 15 years) Not treated with RME Nasal septal angle 0.78 (SD = 1.23)	Occlusal coverage, Hyrax type expander with 2 turns a day for 2–3 weeks	Measured in degrees as angle between the nasal septum midsagittal plane on frontal/PA cephalograms. Measurements taken prior to appliance insertion and after 12 weeks active expansion	No significant positional change in nasal septum from RME

One study [15] reported straightening of the nasal septum by approximately 94 % in the middle and the inferior third of the nasal cavity from RME. Correction in NSD was confirmed by a reduction in the amplitude of septal deviation as measured in millimeters from the midsagittal plane. The included sample were 100 children aged 5 to 9 years. RME was accomplished through hyrax activated twice a day for 15 days.

Another study [21] reported no positional change in the nasal septum from RME. In this study, the nasal septal angle was measured in degrees from the midsagittal plane. The sample consisted of 10 children aged 13–17 years with occlusal coverage hyrax appliance. Expansion protocol in this cohort was twice a day hyrax activation for 2–3 weeks.

Results from MINORS [17] are listed in Table 3. Total scores for both studies were the same. Both included studies stated clear objectives (item 1) and assessed outcomes according to the aim of the study (item 4) with appropriate statistical analysis (item 12). Both studies included patients according to predetermined exclusion/inclusion criteria and measurement protocols (items 3, 4). However, unbiased assessment of outcome variable was not fulfilled by either study (item 5). In addition, patients that could have been lost to follow-up were not reported by either study (item 7). Neither study conducted a prospective sample size calculation from effect size (item 8) or had baseline equivalence of control and treatment groups (item 10). One study [21] recruited the control group from data archives; therefore, the criteria of contemporary control and treatment groups were not fulfilled (item 10).

**Table 3:** Methodological quality assessment of included studies by MINORS

Methodological item	Farronato et al. [15]	Ss score	Altug-Atac et al. [21]	Score
1. A clearly stated aim	Yes	1	Yes	1
2. Inclusion of consecutive patients	Yes	1	Yes	1
3. Prospective collection of data	Yes	1	Yes	1
4. Endpoints appropriate to the aim of the study	Yes	1	Yes	1
5. Unbiased assessment of the study endpoint	No	0	No	0
6. Follow-up period appropriate for the aim of the study	Yes	1	Yes	1
7. Loss to follow-up less than 5 %	Unclear	0.5	Unclear	0.5
8. Prospective calculation of study size	No	0	No	0
9. An adequate control group	No	0	Unclear	0.5
10. Contemporary groups	Yes	1	No	0
11. Baseline equivalence of groups	No	0	Unclear	0.5
12. Adequate statistical analysis	Yes	1	Yes	1
Total score		7.5		7.5

Score key: yes = 1, no = 0, unclear = 0.5

### Discussion

Nasal breathing is a prerequisite for proper growth of the craniofacial complex. Moderate to severe nasal septal deviation (NSD) can cause clinically significant nasal obstruction, resulting in irreversible repercussions on the growth and development of craniofacial structures. The purpose of this systematic review was to investigate the effect of rapid maxillary expansion on nasal septal deviation.

Historically, RME was believed to primarily affect airway function through changes to nasal volume. For example, Haas [13] reported RME resulted in an increased nasal width of 2–4.5 mm with an expansion protocol of 0.4 to 0.5 mm per day for 12 to 27 days in his patient cohort. It was postulated that the alteration in nasal dimensions following RME is related to the lateral movement of the nasal walls [22], increase in the vertical dimension of the nasal cavity secondary to inferior rotation of the palate [12].

Like Haas, many investigators have focused on changes in nasal volume or the secondary effect of changing nasal airflow resistance after RME. These studies yielded inconclusive findings. Some demonstrated positive nasal changes after RME [23, 24], others found no difference [25], while some found such small differences that the clinical relevance was questioned [26, 27]. However, more clinically directed inquiries, such as subjective patient experience [28, 29] and polysomnography changes with sleep apnea [30], have provided growing support to potential functional airway benefits of RME.

Since changes in nasal volume alone seem inconclusive to account for the effects of RME, alternative explanations are now being explored. One of such hypothesis is the effect of RME on the nasal septum. Data from computational fluid dynamic studies that have modeled nasal septal deviations have been valuable in providing [31, 32] comprehensive information on nasal airflow characteristics. These studies concluded that anterior and inferior septal deviations increase nasal resistance more than posterior and superior septal deviations [31, 32]. Consequently, significant septal deviations in the posterior nasal cavity can occur without significant increase in nasal airway resistance. This is explained due to the fact that in healthy nasal passages, majority of the airflow is at the height of the nasal floor and the area between the inferior and middle turbinates, with less than 15 % of nasal airflow at the superior part of the nasal cavity [31]. Rapid maxillary expansion affects nasal airway because it is considered to modify the nasal valve area, which represents the narrowest nasal cross-sectional area [33]. In other words, nasal valve area is likely the greatest contributor to nasal airway resistance during breathing.

Interestingly, patients with maxillary deficiency in the transverse dimension usually also have small nasal cross-sectional areas [34], which can explain the reason for maxillary expansion having a potentially positive effect on the nasal airway. Further investigation of the possibility of RME correcting NSD would be valuable, considering the undesirable sequelae of NSD on nasal breathing, which can consequently affect craniofacial development. In addition, septal cartilage can act as a growth center in early development; its deviation can cause distortion of the maxillary complex toward the deviated side [15].

Although, there are numerous reports of the effects of RME on the nasal airway, only a few of those studies have hypothesized that RME “straightens” the nasal septum, thereby correcting nasal septal deviation [15, 16, 19, 35].

The earliest indication of the potential effect of palatal expansion over the nasal septum anatomy came from Gray [20]. While evaluating 140 cases (mostly between 3 and 14 years of age), he noted a significant improvement in the nasal airway (84 % of the cases), diminution of allergic symptoms (65–70 % of the cases), and infections (87 % of the cases). In addition, a positive psychological benefit (25 % of the cases), as well as dental changes, was noted. The issue with this study was that it is not clear if nasal septum anatomy was assessed after the expansion process. Listed quantified outcomes did not include such assessment. It was implied that the septum deviation was corrected. No specific data analysis was available differentiating changes in children from adolescents.

Another early finding of this effect came also from Gray [16], whereby he noted an improvement in the “curve” of the deviated nasal septum after RME treatment from subjective visualization of posterior anterior radiographs. The sample size in this study consisted of 310 patients ranging from 4 to 24 years of age with majority (86 %) of the patients under the ages of 12. It is not clear, although is likely the case, if the 140 cases from the previous publication [20] were included. Subjective improvement of the nasal airway was reported in these patients with improvement stable at 6 months post expansion. Approximately 80 % of patients reported switching from mouth to nasal breathing post expansion with a significant reduction (roughly 60 %) in colds, sore throats, ear infections, and nasal allergies. No specific data analysis was available differentiating changes in children from adolescents. It was hypothesized that improvement of nasal ventilation from RME prevented dryness and crusting of the nasal mucosa thereby reducing recurrent upper airway infections. Improved ciliary function and normal nasal cycle function were among other benefits purported from increased nasal airflow resulting from RME. However, this study was excluded from our systematic review since the conclusions were based on visual and subjective assessment of X-rays without any objective quantification of change or appropriate statistical analysis. The issue again with this study was that although it is stated that nasal septum improvement was visually assessed, a percentage of correction was not stated.

Finally, Schwarz et al. reported [19] in nine adult patients that underwent surgically assisted maxillary expansion without including nasal septum sectioning failed to notice any nasal septum anatomical change. Coronal tomograms were used to quantify the before and after nasal septum symmetry.

Only two studies [15, 21] were finally included in this systematic review after conducting electronic searches of several databases. Both analyzed the change in nasal septal deviation from RME in two-dimensional coronal views from posterior anterior cephalograms. Farronato et al. [15] recruited 100 growing patients (ages 5–9 years, average 7.62 ± 0.7) presenting with transverse maxillary constriction and measured an increase of 2.3 mm in the width of the nasal cavity and reported 94 % reduction in the septal deviation from RME. The NSD correction was noted in the inferior and middle half of the nasal cavity when compared to a non-expansion control group. Septal correction in this study was measured by placing points on superior, middle, and inferior segments of the septum as visualized from pre and post expansion PA cephalograms. Distances between these landmarks were measured along with maximum amplitude of deviation from an imaginary midline in coronal view. The resulting change was quantified in millimeters and as a percentage. Patient sample in this study had septal deviation of at least 1 mm in the middle/inferior third of the septum as visualized as a deflection in the vertical path from superior to inferior on PA (coronal view) X-rays. However, the results from the other included paper [21] were contradictory to the aforementioned study. The latter study reported no change in NSD from RME in an older cohort of patients (ages 13–17 years).

It has been documented that RME efficacy is greater when done before the growth spurt (2.3 mm) versus at or after the peak growth (1.5 mm) [9]. Incomplete calcification of the midpalatal suture in growing patients translates into ease of displacement of the lateral walls of the nasal cavity [9]. Rapid maxillary expansion treatment in mixed dentition, i.e., prior to midpalatal suture closure has greater improvement in nasal airway resistance due to greater likelihood of skeletal change (nearly 50 %) as opposed to during adolescence when the change is mostly dental. However, to our knowledge, no study has investigated the effect of slow or semi-rapid expansion on nasal airway or structures.

Methodological quality of studies included in this systematic review was analyzed using MINORS checklist. Although, both studies had similar total scores (7.5/12) suggesting moderate level of evidence, there were a few methodological flaws. Both stated clear objectives and assessed outcomes according to the aim of the study; however, the outcome assessor was not blinded, and the reasons for lack of blinding were not mentioned. There was also no prospective sample size calculation, and the reasoning behind this was not elucidated. It is ambiguous whether the baseline characteristics of the control and treatment groups were equivalent. Farronato et al. [15] included a “control” group having no septal deviation and without RME. It would be difficult to ascertain the effect of an intervention such as RME, without a comparable baseline nasal septal deviation in control and treatment groups. Although, the Altug-Atac et al. [21] did report including an age-matched untreated control group, the comparison was historical because the control group was recruited from archived patient database. Furthermore, it is unclear whether the control group and RME group had similar baseline nasal septal deviation for accurate comparison.

Due to lack of literature in this area, it would be beneficial to plan future studies in a preadolescent patient population presenting with transverse maxillary deficiency. The aim of the aforementioned study would be to methodically measure NSD in a three-dimensional view at set landmarks instead of an isolated pre- and post-PA cephalogram image.

### Limitations

The question of whether RME is beneficial in reducing the effect of nasal obstruction from a deviated nasal septum in growing patients has not been intensively investigated.

Except from the hand-search of the references of the identified articles during the phase 1 selection process, no further gray literature was searched. Studies that may have been published in common non-indexed electronic databases may have been missed.

## Conclusions

Thus far, the limited available (moderate risk of bias) evidence suggests a potentially positive effect on the nasal septum asymmetry during childhood, but no significant change in adolescence from RME in patients with NSD. The clinical significance of reported changes in children could be considered questionable.

## Additional file

Additional file 1:
**Database searches.**

## Abbreviations

ACP Journal Club: American College of Physicians Journal Club
CBCT: Cone-beam computed tomography
CCTR: Cochrane Central Register of Controlled Trials
CDSR: Cochrane Database of Systematic Review
CMR: Cochrane Methodology Register
DARE: Database of Abstracts of Reviews of Effects
HTA: Health Technology Assessments
MINORS: Methodological index for non-randomized studies
NHSEED: NHS Economic Evaluation Database
NSD: Nasal septal deviation
RME: Rapid maxillary expansion
