# Supplementary material for: Effect of non-surgical maxillary expansion on the nasal septum deviation: a systematic review
Source: Prog Orthod. 2015 Jun 4;16:15. doi: 10.1186/s40510-015-0084-y (PMC4456578; doi:10.1186/s40510-015-0084-y)
Supplement: Additional file 1: — Database searches. [file 40510_2015_84_MOESM1_ESM.doc]

**Additional file 1: Database Searches**

| **MEDLINE/EMBASE and EMBR** | **Web of Science** |
| --- | --- |
| **MEDLINE**  **1. Exp Nasal Septum/**   1. **Exp maxilla/or exp palatal expansion**   **technique/exp palate/ exp malocclusion**   1. **1 AND 2**   **EMBASE**   1. **Exp nose septum** 2. **Exp maxilla/or exp orthodontics/ or exp malocclusion/ or exp palate/ or exp orthodontic device**   **A. 1 AND 2**  **EMBR**   1. **Nasal septum mp** 2. **Nose mp.** 3. **1 OR 2** 4. **Rapid maxillary expansion.mp** 5. **Rapid palatal expansion.mp** 6. **4 OR 5** 7. **3 AND 6** | Nasal septum* (Topic)  AND  Rapid maxillary expansion* (Topic) |
